# Aflatoxin-B1-Exposure-Induced Hepatic Injury Could Be Alleviated by Polydatin through Reducing Oxidative Stress, Inhibiting Inflammation and Improving Mitophagy

**DOI:** 10.3390/toxics11040309

**Published:** 2023-03-26

**Authors:** Kang Cheng, Jingyi Niu, Xiaotong Zheng, Yining Qiao, Jinyan Zhang, Rui Guo, Guorun Dong, Zhihua Song, Jin Huang, Jinrong Wang, Yong Zhang

**Affiliations:** 1 School of Biological Engineering, Henan University of Technology, Zhengzhou 450001, China; 2 School of International Education, Henan University of Technology, Zhengzhou 450001, China

**Keywords:** aflatoxin B1, polydatin, oxidative stress, inflammation, mitophagy, liver

## Abstract

Aflatoxin B1 (AFB1) is a toxic food/feed pollutant, exerting extensive deleterious impacts on the liver. Oxidative stress and inflammation are considered to be vital contributors to AFB1 hepatotoxicity. Polydatin (PD), a naturally occurring polyphenol, has been demonstrated to protect and/or treat liver disorders caused by various factors through its antioxidant and anti-inflammatory properties. However, the role of PD in AFB1-induced liver injury is still elusive. Therefore, this study was designed to investigate the protective effect of PD on hepatic injury in mice subjected to AFB1. Male mice were randomly divided into three groups: control, AFB1 and AFB1-PD groups. The results showed that PD protected against AFB1-induced hepatic injury demonstrated by the reduced serum transaminase activity, the restored hepatic histology and ultrastructure, which could be attributed to the enhanced glutathione level, the reduced interleukin 1 beta and tumor necrosis factor alpha concentrations, the increased interleukin 10 expression at transcriptional level and the up-regulated mRNA expression related to mitophagy. In conclusion, PD could alleviate AFB1-induced hepatic injury by reducing oxidative stress, inhibiting inflammation and improving mitophagy.

## 1. Introduction

Aflatoxins are highly toxic, mutagenic and carcinogenic compounds mainly produced by *Aspergillus flavus* and *Aspergillus paraiticus*, including aflatoxin B1, B2, G1, G2, M1 and M2, among which aflatoxin B1 (AFB1) represents the strongest toxicity with the most common encounters [1,2,3]. In 1993, AFB1 was classified as a group I carcinogen by the International Agency for Research on Cancer owing to its extremely carcinogenic capability [4]. AFB1 is widespread in the environment, and contaminates human foodstuffs and animal feeds prior to harvest or during storage post-harvest causing public health and food safety problems globally [4]. The consumption of foods/feeds polluted with AFB1 results in damage to the liver evidenced by loss of the normal structure of liver tissues, the enhancing of liver functional enzymes (i.e., aspartate transaminase (AST) and alanine transaminase (ALT)) activities, and obvious cell necrosis and apoptosis, as the liver is the principal target organ where the activation, metabolism and elimination of AFB1 are all carried out [5,6,7]. The metabolic processes of AFB1 in the liver are usually accompanied by oxidative stress and inflammation [8]. Oxidative stress and inflammation are recognized as the fundamental mechanisms of AFB1-induced hepatotoxicity. In vitro and in vivo studies showed that AFB1 exposure impaired the equilibrium of oxidants and antioxidants in the liver, resulting in the oxidative damage of biomolecules such as DNA, proteins and lipids [7,9,10]. An enormous amount of research has reported that AFB1 could induce the inflammatory response and stimulate the synthesis of pro-inflammatory cytokines (e.g., interleukin 1 beta (IL1β) and tumor necrosis factor alpha (TNFα)) by activating signaling pathways and regulating the expression of genes involved in inflammation, especially the classic nucleotide-binding and oligomerization domain-like receptor family pyrin domain-containing 3 (NLRP3) inflammasome pathway [10,11]. In addition, AFB1 disrupts the mitochondrial function and consequently results in excessive ROS production and DNA breakage in the liver, which can aggravate oxidative stress and the inflammatory response [4]. Interestingly, previous studies reported that the impairment of hepatic mitophagy (a selective form of autophagy) in mice exposed to AFB1 may play an important role in mitochondrial damage, which ultimately contributes to liver injury [9]. Thus, it is of significance to explore prevention and/or treatment approaches to minimize AFB1 exposure by modulating redox status, inflammation and mitophagy.

Polydatin (3,4′,5-trihydroxystilbene-3-β-D-glucoside, PD) is a monocrystalline compound isolated from *Polygonum cuspidatum* but is also detected in grape, peanut, hop cones, red wines, hop pellets, cocoa-containing products, chocolate products and many daily diets [12]. It performs various pharmacological activities, including anti-inflammation [13], antioxidant [14], antitumor [15], lipid-lowering [16] and organs/tissues protection effects [12], especially exhibiting strengthened hepatoprotective activity [17]. Accumulating researches have shown that PD can prevent and/or treat liver injury caused by diverse factors, such as alcohol [16], drugs [14], dietary components [13,18] and sulfur mustard [19], which could be attributed to some molecular mechanisms, including the enhancement of autophagy, the inhibition of pro-inflammatory cytokines and lipid accumulation, as well as the increase in antioxidants’ profile (e.g., catalase (CAT), superoxide dismutase (SOD), and glutathione (GSH)). Moreover, in a recent report, PD was demonstrated to protect against mitochondrial dysfunction and NLRP3 inflammasome activation in sepsis-induced acute kidney injury by activating Parkin-dependent mitophagy via the activation of sirtuin 1 (SIRT1) [20]. However, studies investigating the protective effect of PD on AFB1-induced liver injury are absent, and the role of mitophagy is unknown. Therefore, the present study was conducted to test whether PD has the potential to protect against hepatic oxidative stress, inflammation and mitophagy in mice exposed to AFB1 and to explore the possible molecular mechanism.

## 2. Materials and Methods

### 2.1. Animals and Treatment

The experimental protocols were in accordance with guidelines set by Henan University of Technology Institutional Animal Care and Use Committee (Ethic Approval Code: HAUT20211115). A total of 24 male Kunming mice (20 ± 2 g and 6 weeks of age) were purchased from the Huaxing Experimental Animal Farm (Zhengzhou, China). Mice were housed under constant conditions (20–24 °C, 40–60% humidity) with alternative 12 h light–dark cycles. In addition, mice were given access to feed and water. After acclimation of 1 week, mice were randomly assigned into three groups (n = 8) as follows: control (CON), AFB1 and AFB1-PD groups. AFB1 and PD were dissolved in 0.5% dimethyl sulfoxide (DMSO), respectively. Except for the CON group, all mice were orally fed with AFB1 at the dose of 300 μg/kg body weight once a day for 18 days. Mice in the AFB1-PD group were given 100 mg PD/kg body weight/d by oral gavage before AFB1 treatment. Mice in the CON group were orally given 0.5% DMSO, in same volume. The usage dose of AFB1 in the present study was according to the previous investigation [21]. The dose of PD was selected based on the previous study [14]. The PD (≥95%) and AFB1 (≥98%) were purchased from Shanghai Yuanye Bio-Technology Co., Ltd. (Shanghai, China) and Shanghai Macklin Biochemical Co., Ltd. (Shanghai, China), respectively. The DMSO (≥99.5%) was analytical pure and was purchased from Tianjin Kemiou Chemical Reagent Co., Ltd. (Tianjin, China).

### 2.2. Sample Collection

At the end of the experiment, all mice were anesthetized, blood samples were collected from eyeballs. Serum was obtained from blood by centrifugation at 2000× *g* for 15 min at 4 °C, which was stored at −80 °C for further analysis. After blood collection, all mice were killed by cervical dislocation, and necropsy was carried out. The liver samples (left lobe) were rapidly collected and divided into three parts. One-third of the liver tissue was fixed in 4% paraformaldehyde. One-third of the liver tissue was used for the observation of ultrastructure. The remaining one-third part of the liver tissue was immediately snap-frozen in lipid nitrogen and stored at −80 °C for subsequent biochemical analysis.

### 2.3. Haematoxylin and Eosin (H&E) Staining

After being fixed, liver tissues were embedded in paraffin wax. Paraffin-embedded tissues were sectioned at 5 μm thickness. Finally, the slides were stained with H&E using standard procedures and the images were acquired by light microscope (BX51, Tokyo, Japan).

### 2.4. Transmission Electron Microscope

To analyze ultrastructure of liver, approximately 1 mm^3^ liver samples were fixed with 2.5% glutaraldehyde and then post-fixed in 1% osmium tetroxide, dehydrated and embedded in epoxy resin. The ultrathin sections were stained and observed using the transmission electron microscope (Hitachi H-7650, Hitachi Technologies, Tokyo, Japan).

### 2.5. Detection of Serum Transaminase Activities

The activities of serum AST and ALT were analyzed using reagent kits from Nanjing Jiancheng Bioengineering Institute (Nanjing, China). Data were expressed as units per mL serum.

### 2.6. Oxidative Status Analysis

Liver tissues were homogenized in cold 154 mmol/L physiological saline solution (4 °C). The 10% homogenized solution was centrifuged at 4000× *g* for 15 min at 4 °C to obtain the supernatant and immediately stored at −20 °C for further analysis.

Hepatic oxidative status was analyzed according to the previous method [22]. The concentrations of total protein, malondialdehyde (MDA) and glutathione (GSH), the activities of catalase (CAT) and total superoxide dismutase (T-SOD) in the liver samples were detected following the protocols of corresponding commercial kits (Nanjing Jiancheng Bioengineering Institute, Nanjing, China). All results were normalized against total protein level in each sample for inter-sample comparison. The concentration of MDA and GSH were expressed as nmol per milligrams of protein. The activities of CAT and T-SOD were expressed as units per milligrams of protein.

### 2.7. Cytokines Level Measurement

Hepatic TNFα and IL1β contents were measured using enzyme-linked immunosorbent assay test kit (Wuhan Servicebio Technology Co., Ltd., Wuhan, China) according to manufacturer’s protocol. The intra- and inter-assay coefficients of variation were 10% and 15% for TNFα, and 10% and 15% for IL1β, respectively. Data were expressed as picogram per milligrams of protein.

### 2.8. RNA Isolation and Quantitative Real-Time PCR (qRT-PCR) Analysis

Total RNA was isolated from liver samples using the RNA Isolation Reagent (Vazyme Biotech Co., Ltd., Nanjing, China). The concentration and quality of RNA were measured based on the method described by Cheng et al. [23]. After that, the first-strand complementary DNA (cDNA) was synthesized from total RNA with HiScript II Q RT Select SuperMix for qPCR(+gDNA wiper) (Vazyme Biotech Co., Ltd., Nanjing, China). The qRT-PCR analysis was performed using ChamQ SYBR qPCR Master Mix according to the recommended process of the manufacturer (Vazyme Biotech Co., Ltd., Nanjing, China). Each sample was tested in duplicate. The 2^−ΔΔCT^ method was used to calculate the relative mRNA level of target genes after normalization against the reference gene *β-actin*, while the mRNA expression of each target gene of mice in the CON group was assigned as a value of 1. The primers used for qRT-PCR are presented in Table 1.

### 2.9. Statistical Analysis

Statistical analysis was conducted using the statistical software (SPSS version 21.0; SPSS, Inc., Chicago, IL, USA). Data were shown as mean ± standard error (SE). Independent sample *t*-tests were used for comparisons between CON and AFB1 groups as well as AFB1 and AFB1-PD groups. *p*-values less than 0.05 were considered statistically significant.

## 3. Results

### 3.1. PD Alleviates Hepatic Injury in AFB1 Mice

**Figure 1:**
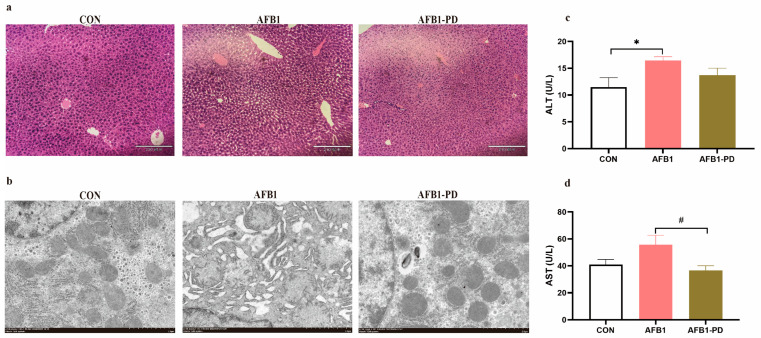
Polydatin (PD) alleviates hepatic injury in aflatoxin B1 (AFB1) mice. (**a**) The HE-stained liver tissues, scale bar = 210 μm; (**b**) the hepatic ultrastructure, scale bar = 2 μm; (**c**) serum alanine transaminase (ALT) activity; (**d**) serum aspartate transaminase (AST) activity. CON, mice are given 0.5% dimethyl sulfoxide; AFB1, mice are given 300 μg AFB1/kg body weight/day; AFB1-PD, mice are given 100 mg PD/kg body weight/day and then exposed to AFB1. Results are represented as mean and standard error (n = 4). * *p* < 0.05 when compared with the CON group, ^#^ *p* < 0.05 when compared with the AFB1 group.

Histological examination and ultrastructure analysis are shown in Figure 1. Vacuolar degeneration of hepatocytes and disorganization of parenchyma and ultrastructural damage including swelling of mitochondria, reduced mitochondrial cristae and endoplasmic reticulum dilatation were exhibited in mice of the AFB1 group, which were improved by PD treatment. In addition, there was an increased serum ALT activity in the AFB1 group compared with that in the CON group (*p* < 0.05). Treatment with PD decreased the activities of AST in the serum of the AFB1-PD group when compared with the CON group (*p* < 0.05).

### 3.2. PD Reduces Hepatic Oxidative Stress in AFB1 Mice

**Figure 2:**
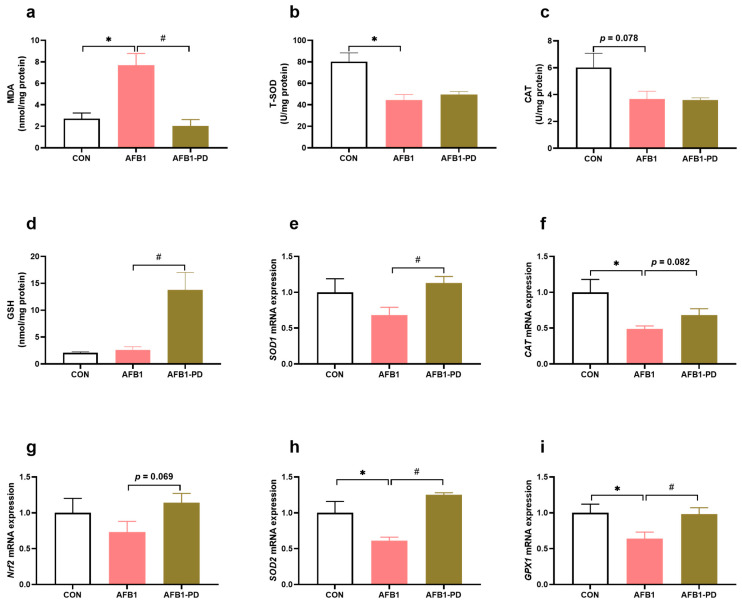
Polydatin (PD) reduces hepatic oxidative stress in aflatoxin B1 (AFB1) mice. (**a**) Malondialdehyde (MDA, n = 6) concentration; (**b**) total superoxide dismutase (T-SOD, n = 6) activity; (**c**) catalase (CAT, n = 6) activity; (**d**) glutathione (GSH, n = 6) level; (**e**–**i**) the mRNA expression of *SOD1*, *CAT*, nuclear factor erythroid 2-related factor 2 (*Nrf2*), *SOD2* and glutathione peroxidase 1 (*GPX1*) (n = 5). CON, mice are given 0.5% dimethyl sulfoxide; AFB1, mice are given 300 μg AFB1/kg body weight/day; AFB1-PD, mice are given 100 mg PD/kg body weight/day and then exposed to AFB1. Results are represented as mean and standard error. * *p* < 0.05 when compared with the CON group, ^#^ *p* < 0.05 when compared with the AFB1 group.

As shown in Figure 2, AFB1 induced an increase in the MDA content (*p* < 0.05), decreased the activities of T-SOD (*p* < 0.05) and CAT (*p* = 0.078), and the expression of the genes glutathione peroxidase 1 (*GPX1*), *SOD2* and *CAT* (*p* < 0.05) in the liver when compared with the CON group. The PD administration decreased MDA accumulation (*p* < 0.05), and increased GSH level (*p* < 0.05) and mRNA expression of nuclear factor erythroid 2-related factor 2 (*Nrf2*, *p* = 0.069), *GPX1* (*p* < 0.05), *SOD1* (*p* < 0.05), *SOD2* (*p* < 0.05) and *CAT* (*p* = 0.082) in the liver of the AFB1-PD group when compared with the AFB1 group.

### 3.3. PD Inhibits Hepatic Inflammation in AFB1 Mice

**Figure 3:**
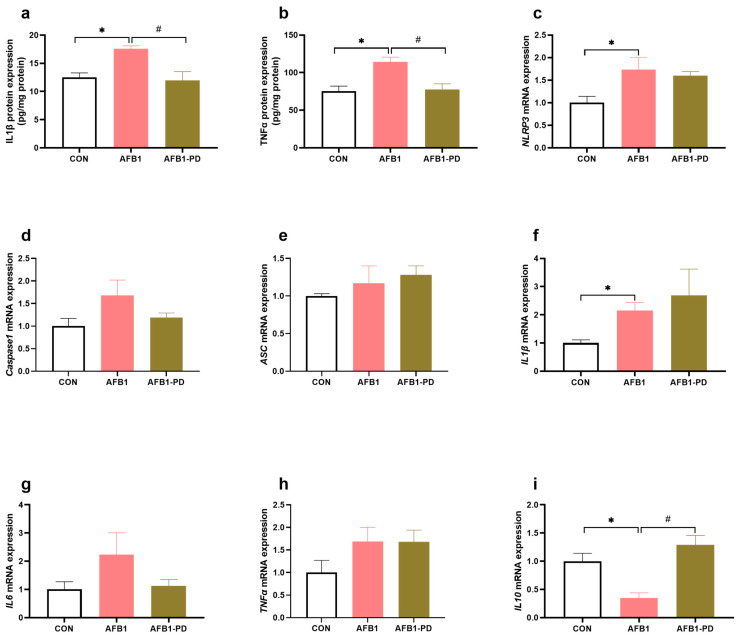
Polydatin (PD) inhibits hepatic inflammation in aflatoxin B1 (AFB1) mice. (**a**,**b**) The protein expression of interleukin 1 beta (IL1β, n = 6) and tumor necrosis factor alpha (TNFα, n = 6); (**c**–**i**) the mRNA expression of nucleotide-binding and oligomerization domain-like receptor family pyrin domain-containing 3 (*NLRP3*), apoptosis-associated speck-like protein (*ASC*), interleukin 6 (*IL6*), *IL1β*, *TNFα* and *IL 10* (n = 5). CON, mice are given 0.5% dimethyl sulfoxide; AFB1, mice are given 300 μg AFB1/kg body weight/day; AFB1-PD, mice are given 100 mg PD/kg body weight/day and then exposed to AFB1. Results are represented as mean and standard error. * *p* < 0.05 when compared with the CON group, ^#^ *p* < 0.05 when compared with the AFB1 group.

In Figure 3, the contents of hepatic IL1β and TNFα were increased (*p* < 0.05) in the AFB1 group compared with the CON group. The administration of PD inhibited the AFB1-induced increase in TNFα and IL1β concentrations in the liver. AFB1-exposed mice had higher (*p* < 0.05) mRNA abundance of *NLRP3* and *IL1β* in the liver, but a lower (*p* < 0.05) *IL10* mRNA expression in the liver, when compared with the CON group. No difference was observed in the expression of genes hepatic *Caspase1*, *ASC*, *IL6* and *TNFα* among the groups (*p* > 0.05). In addition, there was a significant increase in *IL10* gene expression in the AFB1-PD group compared with the AFB1 group (*p* < 0.05).

### 3.4. PD Improves Hepatic Mitophagy in AFB1 Mice

**Figure 4:**
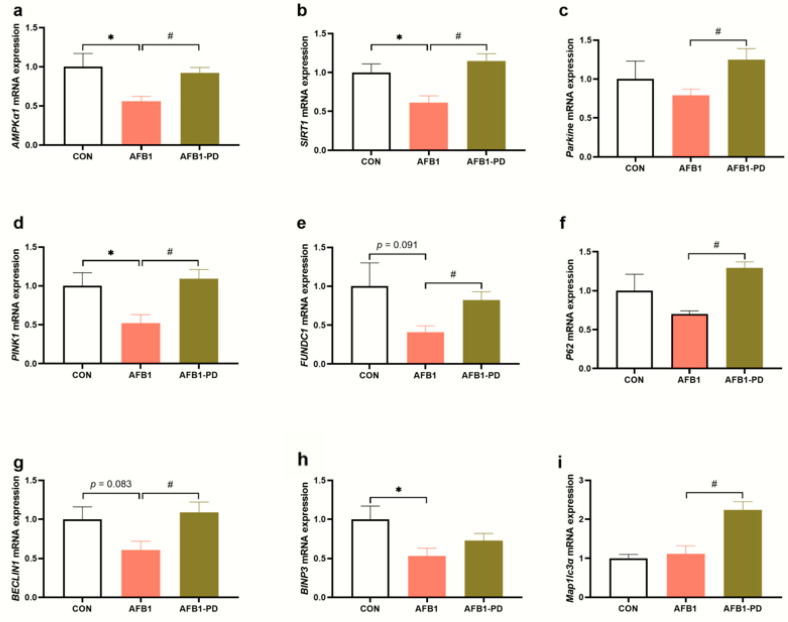
Polydatin (PD) improves hepatic mitophagy in aflatoxin B1 (AFB1) mice. (**a**–**i**) The mRNA expression of protein kinase AMP-activated catalytic subunit alpha 1 (*AMPKα1*), sirtuin 1 (*SIRT1*), *Parkin*, PTEN-induced putative kinase 1 (*PINK1*), FUN14 domain-containing 1 (*FUNDC1*), *P62*, *BECLIN1*, BCL2/adenovirus E1B 19 kDa protein-interacting protein 3 (*BINP3*), microtubule-associated protein 1 light chain 3 alpha (*Map1lc3a*). CON, mice are given 0.5% dimethyl sulfoxide; AFB1, mice are given 300 μg AFB1/kg body weight/day; AFB1-PD, mice are given 100 mg PD/kg body weight/day and then exposed to AFB1. Results are represented as mean and standard error (n = 5). * *p* < 0.05 when compared with the CON group, ^#^ *p* < 0.05 when compared with the AFB1 group.

Compared with the CON group, AFB1 exposure decreased the mRNA abundance of protein kinase AMP-activated catalytic subunit alpha 1 (*AMPKα1*, *p* < 0.05), *SIRT1* (*p* < 0.05), PTEN-induced putative kinase 1 (*PINK1*, *p* < 0.05), FUN14 domain-containing 1 (*FUNDC1*, *p* = 0.091), *BECLIN1* (*p* = 0.083) and BCL2/adenovirus E1B 19 kDa protein-interacting protein 3 (*BINP3*, *p* < 0.05) in the liver (Figure 4). As expected, the mRNA abundances of *AMPKa1*, *SIRT1*, *Parkin, PINK1*, *FUNDC1*, *P62*, *BECLIN1* and microtubule-associated protein 1 light chain 3 alpha in the liver of AFB1-exposed mice were increased by PD treatment (*p* < 0.05).

## 4. Discussion

To the best of our knowledge, this is the first study to report the protective effect of PD on hepatic injury in mice exposed to AFB1. In this study, PD exerted several actions that may contribute to the protective effect, including the increased GSH content, the reduced IL1β and TNFα concentrations, the up-regulated *IL10* gene expression and the improvement in mitophagy, suggesting that PD is a promising compound in terms of its capacity to alleviate AFB1 hepatotoxicity.

Previous researches have indicated that AFB1 exposure leads to liver lesions and dysfunctions and results in the increased activities of circulatory ALT and AST, as well as pathological alterations [5,6,7]. The adverse consequences of AFB1 exposure on liver function and structure were confirmed in this study. Our data also suggested that oral AFB1 administration resulted in significantly elevated serum ALT activity. Moreover, the histopathological changes in the liver (e.g., vacuolar degeneration of hepatocytes and disorganization of parenchyma) and ultrastructural damage were observed, which reinforced the results of the biochemical liver function test in our study. Expectedly, PD treatment ameliorated the hepatic dysfunctions and injury in mice exposed to AFB1, as evidenced by the improved serum transaminase activity and hepatic structure. Similar hepatoprotective effects of PD were also observed in different hepatic injury models, such as alcohol-induced acute liver injury [16], carbon-tetrachloride-induced liver fibrosis [24] and high-fat-diet-induced hepatic steatosis [25], which might be closely associated with its antioxidant and anti-inflammation effects. In addition, recent reports found that Parkin-mediated mitophagy plays an important role in the protective effects of PD against multiple organs injury in a mouse model of sepsis [20,26]. Therefore, we also consider that the protective effect of PD against AFB1-induced hepatic injury in the present study could be partly attributed to the improved redox status, inflammatory response and mitophagy.

Compelling evidence has shown that oxidative stress is involved in the hepatotoxicity of AFB1 [4,27]. During the metabolic process of AFB1 by the hepatic P450 enzymes, excessive reactive oxygen species (ROS) are generated [28]. In addition, mitochondria are highly vulnerable to ROS attacks, and the disruption of the mitochondrial electron transport chain induces electron leakage and ROS production, ultimately resulting in cellular apoptosis and death. Xu et al. [4] reported that the inhibited activities of complexes I-V were associated with ROS produced in the process of AFB1 metabolism, which further contributed to the AFB1-induced oxidative damage. It is well known that oxidative damage can be monitored by determining the levels of antioxidants (e.g., SOD, CAT and GSH) and the biomarkers of lipid peroxidation (e.g., MDA). Similar to previous studies [4,10], the results of this experiment showed that the concentration of MDA was increased, and CAT and T-SOD activities were decreased in the liver of AFB1 mice, indicating that liver oxidative stress existed in mice subjected to AFB1. The inhibited hepatic antioxidant enzyme activity in AFB1 mice in the present study may be associated with its down-regulated mRNA expression and excessive ROS generation. Interestingly, PD treatment improved the hepatic antioxidant capacity of AFB1 mice by reducing MDA generation and increasing GSH content, implying that PD could prevent oxidative stress in the liver of mice exposed to AFB1. GSH can directly bind with AFB1 toxic metabolites to ameliorate AFB1-induced hepatocyte toxicity [29]. However, PD had no effect on antioxidant enzymes activities, i.e., SOD and CAT, which needs to be further explored. Additionally, Nrf2 has been demonstrated to up-regulate the enzymes responsible for detoxification related to GSH synthesis and antioxidant defense systems [30,31]. Many studies have shown that PD induces the activation of the Nrf2 signaling pathway and its targeted detoxification genes expression, in turn relieving hepatic damage in pathological conditions [19,32]. Similarly, in this study, PD increased the *Nrf2* and its target genes’ expression (i.e., *SOD1*, *SOD2*, *CAT* and *GPX1*) in the liver of AFB1 mice. Although the mechanism of PD activating the Nrf2 signaling pathway and enhancing the antioxidant level in the liver of AFB1 mice are unknown, oxidative stress inhibition is a mode of PD action that protects the liver against injury.

Inflammation is also a vital factor for AFB1 hepatotoxicity. A number of danger signals associated with AFB1 metabolites, such as high ROS and toxins, contribute to the inflammatory cascade that triggers cell damage. It is worth noting that the activation of NLRP3 inflammasome plays an essential role in this process. Similar to some previous studies [10,11], the results of this study demonstrated that AFB1 induced the hepatic inflammatory response, as evidenced by the increased concentration of TNFα, the increases in IL1β expression at transcriptional and translational level, and the down-regulated gene expression of *IL10*, which may be associated with the up-regulated mRNA expression of *NLRP3*. Expectedly, PD inhibited the inflammatory cytokines, i.e., TNFα and IL1β production in the liver of AFB1 mice. Our findings suggest that PD attenuated the inflammatory response in the mice subjected to AFB1. However, PD treatment did not affect the genes’ expression of NLRP3 inflammasome and its target pro-inflammatory cytokines. Since ROS is an inflammation activator, we speculate that PD may inhibit the inflammatory response in the liver of AFB1 mice via the suppression of ROS production independent of the NLRP3 inflammasome pathway, but the underlying mechanism needs further investigation.

Mitophagy, a selective autophagy, can effectively remove damaged mitochondria to prevent the accumulation of potentially cytotoxic mitochondrial byproducts in the cytosol, and thus protect the liver from tissue damage [33]. Very recently, mitophagy played a crucial role in attenuating AFB1-induced liver injury partly by counteracting oxidative stress and resisting NLRP3 inflammasome activation [9]. Mitophagy may occur via two different signal pathways, the PINK1/Parkin and autophagy receptor protein (e.g., BNIP3, FUNDC1, and Beclin1) dependent pathways [34]. In the present study, mitophagy-related genes, such as *PINK1*, *BINP3*, *FUNDC1* and *BECLIN1*, were down-regulated in the liver of AFB1 mice, indicating that AFB1 may suppress hepatic mitophagy. However, Wang et al. [9] reported that AFB1 challenge induced mitophagy in the liver of C57BL/6N mice by enhancing Parkin and PINK1 expression. Although Parkin/PINK1-mediated mitophagy was increased in the previous study as an adaptation response, the liver injury induced by AFB1 still occurred in these mice. This discrepancy in the results of AFB1 treatment could be attributed to the animal species. Previous studies explored the regulatory role of PD in mitophagy. For example, Li et al. [26] reported that PD promoted Parkin translocation to the mitochondria and facilitated mitophagy in acute-respiratory-distress-syndrome-challenged mice and lipopolysaccharide-treated Beas-2B cells. Similarly, Gao et al. [20] showed that PD can alleviate sepsis-induced acute kidney injury through the suppression of NLRP3 inflammasome activation by up-regulating Parkin-dependent mitophagy. Furthermore, they demonstrated that the promoting effect of PD on Parkin-dependent mitophagy is associated with the activation of SIRT1. As expected, PD up-regulated the hepatic mitophagy-related genes’ expression in AFB1 mice, which may be attributed to the up-regulation of *SIRT1* and *AMPKα1* mRNA. SIRT1, a highly conserved histone deacetylases, has been implicated in the destruction of damaged mitochondria via mitophagy [35]. A previous study reported that the loss of SIRT1 could delay Parkin translocation to the mitochondria and reduce mitophagy in the luminal epithelium in human prostate cancer [36]. AMPK is a sensor of energy balance in metabolic stress and could up-regulate the transcriptional expression of mitophagy-related genes, such as BINP3 and Parkin. This phenomenon has been demonstrated in various organs disorders, including diabetic nephropathy [37], intestinal oxidative damage [38] and muscle atrophy [39]. The interlinked signaling pathways of mitophagy in response to PD administration would offer new targets for preventive or therapeutic methods of liver protection in the setting of AFB1 exposure. In addition, the enhanced hepatic mitophagy of AFB1-challenged mice receiving PD administration may be related to both the improved redox status and the suppressed inflammatory response.

## 5. Conclusions

**Figure 5:**
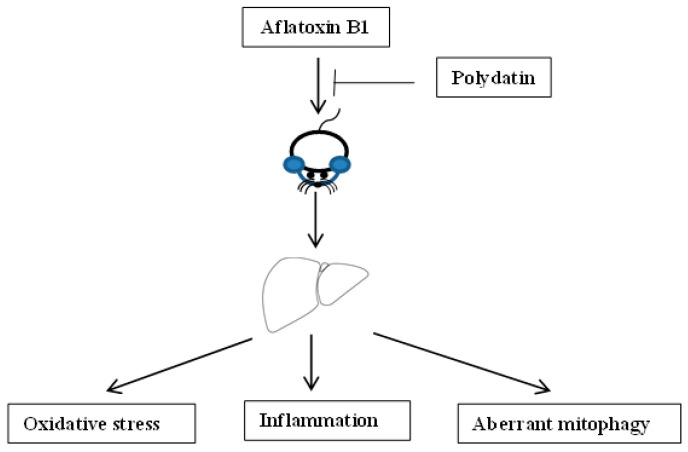
Schematic representation of the protective effect exerted by polydatin on hepatic injury induced by aflatoxin B1.

In conclusion, AFB1 exposure induced hepatic injury, oxidative stress, inflammation and aberrant mitophagy. However, PD administration attenuated the hepatic damage induced by AFB1, which might be associated with the improvement in the redox and inflammatory status and mitophagy, i.e., the enhanced GSH level, the reduced IL1β and TNFα concentrations, the increased IL10 expression at transcriptional level and the up-regulated mRNA expression related to mitophagy (Figure 5). Our study suggests that PD could have potential for further development into a promising therapeutic agent for aflatoxicosis.

## Figures and Tables

**Table 1:** Sequences for real-time PCR primers.

Genes	Forward Primer (5′-3′)	Reverse Primer (5′-3′)
*SIRT1*	ACTGGAGCTGGGGTTTCTGT	CACAGAGACGGCTGGAACTG
*AMPK* *α* *1*	ACCTGAGAACGTCCTGCTTG	GGCCTGCGTACAATCTTCCT
*PINK1*	TGTGGAATATCTCGGCAGGT	GATGTTAGGGTGTGGGGCAA
*Parkin*	GCCAGAGGTCCAGCAGTTAAA	CACCACTCATCCGGTTTGGA
*BNIP3*	TTCTCACTGTGACAGCCCAC	GGTCGACTTGACCAATCCCA
*FUNDC1*	CTTCTTCTTCAGGCAACAGACT	GCAAAAAGCCTCCCACGAAG
*P62*	GGACAGCCAGAGGAACAGAT	CTCAATCAGCCGGGGATCAG
*Map1lc3* *α*	TTGGTCAAGATCATCCGGCG	GAAGGTTTCTTGGGAGGCGT
*BECLIN1*	GCCTCTGAAACTGGACACGA	TGTAGACATCATCCTGGCTGG
*Nrf2*	GCCCTCAGCATGATGGACTT	AACTTGTACCGCCTCGTCTG
*GPX1*	AGTCCACCGTGTATGCCTTC	CCTCAGAGAGACGCGACATT
*SOD1*	CATGGCGATGAAAGCGGTG	GCACTGGTACAGCCTTGTGTA
*SOD2*	AACGCCACCGAGGAGAAGTA	TCCAGCAACTCTCCTTTGGGT
*CAT*	TGATCTGACCAAGGTTTGGC	AGTGTCCGGGTAGGCAAAAA
*NLRP3*	TCTCCCGCATCTCCATTTGT	CTGTCCCGCATTTTAGTCCG
*Caspase1*	CAGGAGGGAATATGTGGG	CACCTTGGGCTTGTCTTT
*ASC*	TGACAGTGCAACTGCGAGAA	GTGAGCTCCAAGCCATACGA
*IL10*	CCAAGGTGTCTACAAGGCCA	GCTCTGTCTAGGTCCTGGAGT
*IL6*	CTCCCAACAGACCTGTCTATAC	CCATTGCACAACTCTTTTCTCA
*IL1* *β*	GTCTTTCCCGTGGACCTTC	ATCTCGGAGCCTGTAGTGC
*TNF* *α*	GCGACGTGGAACTGGCAGAAG	GCCACAAGCAGGAATGAGAAGAGG
*β* *-actin*	TATAAAACCCGGCGGCGCA	GTCATCCATGGCGAACTGGTG

*AMPKα1*, protein kinase AMP-activated catalytic subunit alpha 1; *ASC*, apoptosis-associated speck-like protein; *BNIP3*, BCL2/adenovirus E1B 19 kDa protein-interacting protein 3; *CAT*, catalase; *FUNDC1*, FUN14 domain-containing 1; *GPX1*, glutathione peroxidase 1; *IL1β*, interleukin 1 beta; *IL6*, interleukin 6; *IL10*, interleukin 10; *Map1lc3*α, microtubule-associated protein 1 light chain 3 alpha; *NLRP3*, nucleotide-binding and oligomerization domain-like receptor family pyrin domain-containing 3; *Nrf2*, nuclear factor erythroid 2-related factor 2; *PINK1*, PTEN-induced putative kinase 1; *P62*; *SIRT1*, sirtuin 1; *SOD1*, superoxide dismutase 1; *SOD2*, superoxide dismutase 2; *TNFα*, tumor necrosis factor alpha; *β-actin*, beta actin.

## Data Availability

Data will be made available on request.

## References

[1] Yunus A.W., Razzazi-Fazeli E., Bohm J. (2011). Aflatoxin B1 in affecting broiler’s performance, immunity, and gastrointestinal tract: A review of history and contemporary issues. Toxins.

[2] Ji Y., Nyamagoud S.B., SreeHarsha N., Mishra A., Gubbiyappa S.K., Singh Y. (2020). Sitagliptin protects liver against aflatoxin B1-induced hepatotoxicity through upregulating Nrf2/ARE/HO-1 pathway. BioFactors.

[3] Damiano S., Jarriyawattanachaikul W., Girolami F., Longobardi C., Nebbia C., Andretta E., Lauritano C., Dabbou S., Avantaggiato G., Schiavone A. (2022). Curcumin Supplementation Protects Broiler Chickens Against the Renal Oxidative Stress Induced by the Dietary Exposure to Low Levels of Aflatoxin B1. Front. Vet. Sci..

[4] Xu F., Li Y., Cao Z., Zhang J., Huang W. (2021). AFB1-induced mice liver injury involves mitochondrial dysfunction mediated by mitochondrial biogenesis inhibition. Ecotoxicol. Environ. Saf..

[5] Muhammad I., Wang X., Li S., Li R., Zhang X. (2018). Curcumin confers hepatoprotection against AFB1-induced toxicity via activating autophagy and ameliorating inflammation involving Nrf2/HO-1 signaling pathway. Mol. Biol. Rep..

[6] Taranu I., Hermenean A., Bulgaru C., Pistol G.C., Ciceu A., Grosu I.A., Marin D.E. (2020). Diet containing grape seed meal by-product counteracts AFB1 toxicity in liver of pig after weaning. Ecotoxicol. Environ. Saf..

[7] Wang X., He Y., Tian J., Muhammad I., Liu M., Wu C., Xu C., Zhang X. (2021). Ferulic acid prevents aflatoxin B1-induced liver injury in rats via inhibiting cytochrome P450 enzyme, activating Nrf2/GST pathway and regulating mitochondrial pathway. Ecotoxicol. Environ. Saf..

[8] Pauletto M., Tolosi R., Giantin M., Guerra G., Barbarossa A., Zaghini A., Dacasto M. (2020). Insights into Aflatoxin B1 Toxicity in Cattle: An In Vitro Whole-Transcriptomic Approach. Toxins.

[9] Wang Q., Jia F., Guo C., Wang Y., Zhang X., Cui Y., Song M., Cao Z., Li Y. (2022). PINK1/Parkin-mediated mitophagy as a protective mechanism against AFB1-induced liver injury in mice. Food Chem. Toxicol..

[10] Wang Y., Liu F., Liu M., Zhou X., Wang M., Cao K., Jin S., Shan A., Feng X. (2022). Curcumin mitigates aflatoxin B1-induced liver injury via regulating the NLRP3 inflammasome and Nrf2 signaling pathway. Food Chem. Toxicol..

[11] Jin S., Yang H., Wang Y., Pang Q., Jiao Y., Shan A., Feng X. (2021). Dietary Curcumin Alleviated Aflatoxin B1-Induced Acute Liver Damage in Ducks by Regulating NLRP3-Caspase-1 Signaling Pathways. Foods.

[12] Du Q.H., Peng C., Zhang H. (2013). Polydatin: A review of pharmacology and pharmacokinetics. Pharm. Biol..

[13] Zhao X.J., Yu H.W., Yang Y.Z., Wu W.Y., Chen T.Y., Jia K.K., Kang L.L., Jiao R.Q., Kong L.D. (2018). Polydatin prevents fructose-induced liver inflammation and lipid deposition through increasing miR-200a to regulate Keap1/Nrf2 pathway. Redox Biol..

[14] Liu Y.H., Huang Q.H., Wu X., Wu J.Z., Liang J.L., Lin G.S., Xu L.Q., Lai X.P., Su Z.R., Chen J.N. (2018). Polydatin protects against acetaminophen-induced hepatotoxicity in mice via anti-oxidative and anti-apoptotic activities. Food Funct..

[15] Xu G., Kuang G., Jiang W., Jiang R., Jiang D. (2016). Polydatin promotes apoptosis through upregulation the ratio of Bax/Bcl-2 and inhibits proliferation by attenuating the β-catenin signaling in human osteosarcoma cells. Am. J. Transl. Res..

[16] Lai Y., Zhou C., Huang P., Dong Z., Mo C., Xie L., Lin H., Zhou Z., Deng G., Liu Y. (2018). Polydatin alleviated alcoholic liver injury in zebrafish larvae through ameliorating lipid metabolism and oxidative stress. J. Pharmacol. Sci..

[17] Tang D., Zhang Q., Duan H., Ye X., Liu J., Peng W., Wu C. (2022). Polydatin: A Critical Promising Natural Agent for Liver Protection via Antioxidative Stress. Oxid. Med. Cell. Longev..

[18] Chen X., Chan H., Zhang L., Liu X., Ho I.H.T., Zhang X., Ho J., Hu W., Tian Y., Kou S. (2019). The phytochemical polydatin ameliorates non-alcoholic steatohepatitis by restoring lysosomal function and autophagic flux. J. Cell. Mol. Med..

[19] Zhang H., Chen Y., Pei Z., Gao H., Shi W., Sun M., Xu Q., Zhao J., Meng W., Xiao K. (2019). Protective effects of polydatin against sulfur mustard-induced hepatic injury. Toxicol. Appl. Pharmacol..

[20] Gao Y., Dai X., Li Y., Li G., Lin X., Ai C., Cao Y., Li T., Lin B. (2020). Role of Parkin-mediated mitophagy in the protective effect of polydatin in sepsis-induced acute kidney injury. J. Transl. Med..

[21] Huang L., Zhao Z., Duan C., Wang C., Zhao Y., Yang G., Gao L., Niu C., Xu J., Li S. (2019). Lactobacillus plantarum C88 protects against aflatoxin B1-induced liver injury in mice via inhibition of NF-κB–mediated inflammatory responses and excessive apoptosis. BMC Microbiol..

[22] El-Aarag B., Magdy M., AlAjmi M.F., Khalifa S.A.M., El-Seedi H.R. (2019). Melittin Exerts Beneficial Effects on Paraquat-Induced Lung Injuries in Mice by Modifying Oxidative Stress and Apoptosis. Molecules.

[23] Cheng K., Song Z., Li S., Yan E., Zhang H., Zhang L., Wang C., Wang T. (2019). Effects of resveratrol on intestinal oxidative status and inflammation in heat-stressed rats. J. Therm. Biol..

[24] Zhao X., Li R., Liu Y., Zhang X., Zhang M., Zeng Z., Wu L., Gao X., Lan T., Wang Y. (2017). Polydatin protects against carbon tetrachloride-induced liver fibrosis in mice. Arch. Biochem. Biophys..

[25] Zhang Q., Tan Y., Zhang N., Yao F. (2015). Polydatin supplementation ameliorates diet-induced development of insulin resistance and hepatic steatosis in rats. Mol. Med. Rep..

[26] Li T., Liu Y., Xu W., Dai X., Liu R., Gao Y., Chen Z., Li Y. (2019). Polydatin mediates Parkin-dependent mitophagy and protects against mitochondria-dependent apoptosis in acute respiratory distress syndrome. Lab. Investig..

[27] Gao X., Xu J., Jiang L., Liu W., Hong H., Qian Y., Li S., Huang W., Zhao H., Yang Z. (2021). Morin alleviates aflatoxin B1-induced liver and kidney injury by inhibiting heterophil extracellular traps release, oxidative stress and inflammatory responses in chicks. Poult. Sci..

[28] Shen H.M., Shi C.Y., Shen Y., Ong C.N. (1996). Detection of elevated reactive oxygen species level in cultured rat hepatocytes treated with aflatoxin B1. Free Radical Biol. Med..

[29] Hayes J.D., Pulford D.J., Ellis E.M., McLeod R., James R.F.L., Seidegård J., Mosialou E., Jernström B., Neal G.E. (1998). Regulation of rat glutathione S-transferase A5 by cancer chemopreventive agents: Mechanisms of inducible resistance to aflatoxin B1. Chem.-Biol. Interact..

[30] Soetikno V., Sari F.R., Lakshmanan A.P., Arumugam S., Harima M., Suzuki K., Kawachi H., Watanabe K. (2013). Curcumin alleviates oxidative stress, inflammation, and renal fibrosis in remnant kidney through the Nrf2–keap1 pathway. Mol. Nutr. Food Res..

[31] Muhammad I., Wang H., Sun X., Wang X., Han M., Lu Z., Cheng P., Hussain M.A., Zhang X. (2018). Dual Role of Dietary Curcumin Through Attenuating AFB1-Induced Oxidative Stress and Liver Injury via Modulating Liver Phase-I and Phase-II Enzymes Involved in AFB1 Bioactivation and Detoxification. Front. Pharmacol..

[32] Huang Q.H., Xu L.Q., Liu Y.H., Wu J.Z., Wu X., Lai X.P., Li Y.C., Su Z.R., Chen J.N., Xie Y.L. (2017). Polydatin Protects Rat Liver against Ethanol-Induced Injury: Involvement of CYP2E1/ROS/Nrf2 and TLR4/NF-κB p65 Pathway. Evid. Based Complement. Alternat. Med..

[33] Lee S., Kim J.S. (2014). Mitophagy: Therapeutic Potentials for Liver Disease and Beyond. Toxicol. Res..

[34] Ma X., McKeen T., Zhang J., Ding W.X. (2020). Role and Mechanisms of Mitophagy in Liver Diseases. Cells.

[35] Yoshii S.R., Mizushima N. (2015). Autophagy machinery in the context of mammalian mitophagy. Biochim. Biophys. Acta.

[36] Di Sante G., Pestell T.G., Casimiro M.C., Bisetto S., Powell M.J., Lisanti M.P., Cordon-Cardo C., Castillo-Martin M., Bonal D.M., Debattisti V. (2015). Loss of Sirt1 Promotes Prostatic Intraepithelial Neoplasia, Reduces Mitophagy, and Delays Park2 Translocation to Mitochondria. Am. J. Pathol..

[37] Liu Z., Liu H., Xiao L., Liu G., Sun L., He L. (2019). STC-1 ameliorates renal injury in diabetic nephropathy by inhibiting the expression of BNIP3 through the AMPK/SIRT3 pathway. Lab. Investig..

[38] Cao S., Xiao H., Li X., Zhu J., Gao J., Wang L., Hu C. (2021). AMPK-PINK1/Parkin Mediated Mitophagy Is Necessary for Alleviating Oxidative Stress-Induced Intestinal Epithelial Barrier Damage and Mitochondrial Energy Metabolism Dysfunction in IPEC-J2. Antioxidants.

[39] Bak D.H., Na J., Im S.I., Oh C.T., Kim J.Y., Park S.K., Han H.J., Seok J., Choi S.Y., Ko E.J. (2019). Antioxidant effect of human placenta hydrolysate against oxidative stress on muscle atrophy. J. Cell. Physiol..

